# Is Visual Perceptual Narrowing an Obligatory Developmental Process?

**DOI:** 10.3389/fpsyg.2018.02326

**Published:** 2018-11-23

**Authors:** Andrea Sorcinelli, Athena Vouloumanos

**Affiliations:** Department of Psychology, New York University, New York, NY, United States

**Keywords:** perceptual narrowing, perceptual development, face perception, eye-tracking, conspecific, monkey

## Abstract

Perceptual narrowing, or a diminished perceptual sensitivity to infrequently encountered stimuli, sometimes accompanied by an increased sensitivity to frequently encountered stimuli, has been observed in unimodal speech and visual perception, as well as in multimodal perception, leading to the suggestion that it is a fundamental feature of perceptual development. However, recent findings in unimodal face perception suggest that perceptual abilities are flexible in development. Similarly, in multimodal perception, new paradigms examining temporal dynamics, rather than standard overall looking time, also suggest that perceptual narrowing might not be obligatory. Across two experiments, we assess perceptual narrowing in unimodal visual perception using remote eye-tracking. We compare adults’ looking at human faces and monkey faces of different species, and present analyses of standard overall looking time and temporal dynamics. As expected, adults discriminated between different human faces, but, unlike previous studies, they also discriminated between different monkey faces. Temporal dynamics revealed that adults more readily discriminated human compared to monkey faces, suggesting a processing advantage for conspecifics compared to other animals. Adults’ success in discriminating between faces of two unfamiliar monkey species calls into question whether perceptual narrowing is an obligatory developmental process. Humans undoubtedly diminish in their ability to perceive distinctions between infrequently encountered stimuli as compared to frequently encountered stimuli, however, consistent with recent findings, this narrowing should be conceptualized as a refinement and not as a loss of abilities. Perceptual abilities for infrequently encountered stimuli may be detectable, though weaker compared to adults’ perception of frequently encountered stimuli. Consistent with several other accounts we suggest that perceptual development must be more flexible than a perceptual narrowing account posits.

## Introduction

Humans become less sensitive to distinctions and cross-modal correspondences between less frequently encountered stimuli over time, in a developmental process termed perceptual narrowing. The first evidence for perceptual narrowing came from speech perception: younger infants could discriminate between native and non-native speech sounds, whereas older infants became less sensitive to non-native speech (Werker and Tees, 1984; Kuhl et al., 1992). But it was evidence from face perception which suggested that perceptual narrowing was not limited to speech perception, and instead was a fundamental and perhaps obligatory developmental process. Younger infants could discriminate between different human and different monkey faces but older infants and adults were only able to discriminate human faces and not monkey faces (Pascalis and Bachevalier, 1998; Pascalis et al., 2002). Multimodal perception provided further evidence that perceptual narrowing may be a general developmental process, with younger infants appearing to match unfamiliar sounds and sights, like monkey calls to monkey faces, and non-native speech to dynamic faces, but older infants failing to match (Lewkowicz and Ghazanfar, 2006; Pons et al., 2009). Perceptual narrowing has been suggested to be a fundamental and general aspect of perceptual development as it has been observed in multiple modalities and under varied conditions.

Recent findings, however, have cast doubt on the robustness of perceptual narrowing as a ubiquitous developmental phenomenon particularly in unimodal visual perception and multimodal perception. In vision, 9 and 12 months old, who fail to discriminate between infrequently encountered monkey faces in a standard paradigm, are able to discriminate those same faces after increased exposure (Pascalis et al., 2005) or familiarization time (Fair et al., 2012). Additionally, 9 months old discriminate between both frequently encountered same-race faces and infrequently encountered other-race faces, though the ability to discriminate infrequent faces appears to emerge later in development (Chien et al., 2016). In multimodal perception, 6 months old can match an unfamiliar non-native language with a face from another race, but not their familiar native language with a face of their own race (Uttley et al., 2013), and 12 months old can match unfamiliar non-native speech with a dynamic speaking face but not familiar native speech with a corresponding dynamic speaking face (Kubicek et al., 2014), both of which are inconsistent with a perceptual narrowing account. Perceptual development may be more flexible than a perceptual narrowing account posits.

At the same time, there is evidence for perceptual learning in early development (e.g., Fair et al., 2012; Chien et al., 2016), in which processing of frequently encountered native speech sounds improves, even as perception of infrequently encountered sounds and sights may or may not diminish. This developmental pattern has been observed for non-native speech sounds (Kuhl et al., 2006; Burns et al., 2007) and may parallel findings in the broader animal literature in which several species are found to perceive both same-species and other-species signals but show a processing advantage for members of their own species (Kendrick et al., 2001; Peirce et al., 2001; Musser et al., 2014; Araki et al., 2016)

Some previous evidence consistent with perceptual narrowing may have been an artifact of tasks or analysis techniques that were ill-suited to capture complex perception in older infants. For example, in the multimodal matching literature, tasks typically rely on overall looking time across the entire length of manipulation trials (e.g., Werker and Tees, 1984; Lewkowicz and Ghazanfar, 2006; Uttley et al., 2013; Goddard et al., 2016) which might be more appropriate for younger infants, and less appropriate for older infants and adults. In a recent study on matching multimodal audio-visual stimuli (primate faces and vocalizations) in 12 and 18 months old infants and adults, overall looking time and dynamic time course of fixations yielded different results: overall looking time showed apparent failures to match, whereas dynamic time course of fixations revealed successful matching (unpublished data from our laboratory). Thus, it is possible that overall patterns of looking may not be sensitive to the temporal dynamics of older participants’ looking behaviors and that time course analytic approaches may reveal evidence of sensitivity to infrequently encountered stimuli where previous research has not. Evidence of discriminating infrequently encountered stimuli in unimodal visual perception – such as recognizing own- and other-species primate faces – would suggest that perceptual narrowing might not be obligatory in perceptual development.

In a now classic finding in perceptual development, showing that perceptual narrowing extended beyond speech to face perception, young infants discriminated between different human and different monkey faces, but older infants and adults discriminated only human, and not monkey faces (Pascalis and Bachevalier, 1998; Pascalis et al., 2002). Critically in this classic study, failures to discriminate monkey faces were obtained using standard overall looking time analyses, which may not be the most sensitive analytic approach, particularly for adults. Given that dynamic time course of fixations may be more sensitive for assessing multimodal perceptual matching for older participants, we put perceptual narrowing for faces to the test: we examined adults’ discrimination of faces of different primate species, by analyzing the time course of fixations in addition to overall looking, by replicating the conditions of Pascalis et al. (2002) and by using power analyses to determine appropriate sample size. In the present studies, we sought to obtain a sample large enough to detect an effect of medium size at 80% power (*N* = 25). Across two experiments, we compare human adults’ looking at human faces with two different species of monkey faces, rhesus macaques and Barbary macaques, using remote eye-tracking, and analyze overall looking time and the time course of fixations, to assess perceptual narrowing in unimodal visual perception.

## Experiment 1: Rhesus Monkeys

### Materials and Methods

#### Participants

Participants were 24 undergraduate students (*M* age = 19.4 years, *SD* = 2.0; 17 females) at New York University. All participants gave informed consent and received academic credit for their participation. All participants had normal or corrected-to-normal vision (20/20). We excluded one additional participant for failure to meet eye-tracking inclusion criteria (see Eye-Tracking Data Reduction and Inclusion Criteria below).

#### Procedure

Participants viewed human and rhesus monkey faces in a Visual Paired Comparison (VPC) paradigm following Pascalis et al. (2002; Supplementary Materials). Participants were seated approximately 60 cm in front of a monitor displaying photos of faces, while we tracked their gaze using an eye-tracker. Participants were instructed to watch the display “as if they were watching TV,” following Pascalis et al. (2002). First participants’ attention was drawn to the center of the screen by a telescoping bulls eye and attention-getting sounds. Then participants were presented with sets of trials that consisted of one familiarization trial and two test trials, following Pascalis et al. (2002). During the familiarization trial, a single face (either human or monkey) was presented in the center of the screen for 5 s, followed by a 5 s delay with a blank screen. During the first test trial, participants saw the familiar face and a novel face of the same species, presented side-by-side and separated by a 12 cm gap for 5 s. During the second test trial, participants were presented with the same familiar and novel faces having switched sides. Trial sets were followed by a 30 s delay before the presentation of attention-getting telescoping bulls-eye and the start of the next trial set. Participants always completed three sets of monkey trials (one familiarization and two test trials) prior to four sets of human trials, following Pascalis et al. (2002; Supplemental Materials). The data are openly available at https://figshare.com/s/f842956676996db1fc2d.

#### Stimuli

Stimuli were 14 color images of human and rhesus monkey (*Macaca mulatta)* faces. Human and rhesus monkey faces measured 15 cm/14.2 degrees of visual angle vertically and 10 cm/9.5 degrees of visual angle horizontally at a viewing distance of 60 cm. Oval shaped AOIs covering each face during experimental trials were 327756 pixels.

**FIGURE 1:**
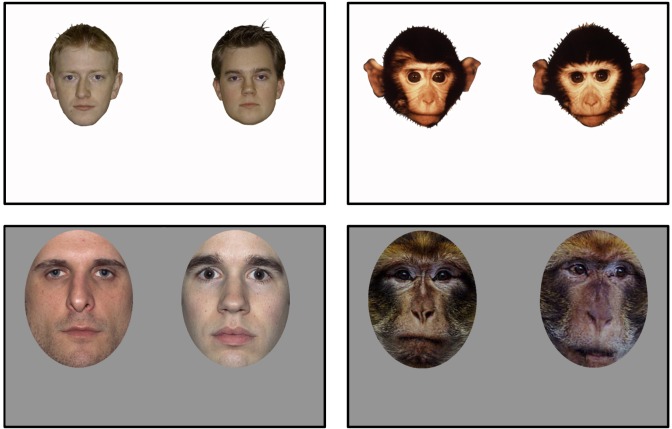
Stimuli. Sample human and monkey faces from Experiment 1 (top; humans and rhesus monkeys) and Experiment 2 (bottom; humans and Barbary monkeys). From Pascalis et al. (2002). Reprinted with permission from AAAS.

Stimuli were those used in Pascalis et al. (2002) and were obtained courtesy of Dr. Pascalis (see Figure 1).

#### Apparatus

During testing, participants sat approximately 60 cm from a 29 cm × 47 cm screen in a sound-attenuated room. At this viewing distance, the entire stimulus display measured 27.8° vertical and 43.1° horizontal visual angle. We used the SensoMotoric Instruments (SMI) RED or RED-m infrared eye-tracker system (SensoMotoric Instruments GmbH, Tetlow, Germany^1^) to measure pupil and corneal reflection, sampled at 60 or 120 Hz. Calibration and stimulus presentation were controlled by SMI IView X^TM^ (Version 2.8, 2014) and Experiment Center^TM^ (Version 3.4, 2014). We obtained a 5-point calibration before beginning data collection and immediately thereafter assessed the accuracy of the calibration by presenting a telescoping bulls-eye in tandem with attention-getting sounds in the center of the display. If participants’ gaze was not within approximately one degree of visual angle of the bulls-eye, we repeated the procedure until an accurate calibration was obtained. Prior to the first and in between each subsequent experimental trial, participants’ attention was drawn to the center of the screen using the same telescoping bulls-eye. The eye-tracker software used a four-point validation at the end of testing to measure any drift in the precision of the tracking after the initial calibration.

#### Eye-Tracking Data Reduction and Inclusion Criteria

After collection, we processed eye-tracking data using SMI BeGaze^TM^ Eye-Tracking Analysis Software (Version 3.4, 2014). We defined two *a priori* areas of interest (AOIs) for the experimental trials that were oval shaped and covered each face (327756 pixels). Areas of interest were not defined for the familiarization trials before each test trial. We calculated fixation lengths and locations by filtering raw data and using predetermined criteria (80 ms, 100 pixels of dispersion) for each individual participant using BeGaze^TM^ Software. We *a priori* defined inclusion criteria as contributing eye-tracking fixations for more than half of a trial, for 50% or more of trials.

### Results

#### Overall Looking at Human and Monkey Faces

**FIGURE 2:**
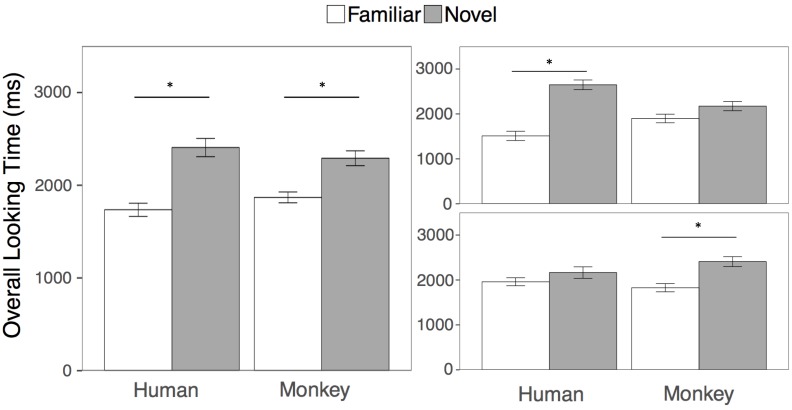
Results. Overall looking times to the Familiar (white bars) and Novel faces (gray bars) in the human and rhesus monkey conditions averaged across both test trials (left), and separately for the first (top right) and second (bottom right) test trials. Error bars represent the standard error of the mean. ^∗^*p* < 0.01.

The goal of the overall looking time analysis was to be directly comparable with the results of Pascalis et al. (2002). Following that approach, we first compared looking at the familiar and novel human and monkey faces in two separate paired samples *t*-tests collapsed across both test trials. Across test trials 1 and 2, participants looked overall longer at the novel compared to the familiar face for humans (*t*(23) = 4.93, *p* < 0.001, *m*_novel_ = 2407 ms, *sd* = 479; *m*_familiar_ = 1735 ms, *sd* = 349, 95, % *CI*_diff_ = [389, 953], *d* = 1.00) and for rhesus monkeys (*t*(23) = 3.47, *p* = 0.002, *m*_novel_ = 2291 ms, *sd* = 392; *m*_familiar_ = 1869 ms, *sd* = 285, 95, % *CI*_diff_ = [170, 674], *d* = 0.71). See Figure 2 for overall looking times to the faces.

However, we wanted to also examine whether that adults looking to the familiar and novel primate faces might differ depending on trial or species, using standard analyses. We then conducted a 2 (Species: Human, Monkey) × 2 (Familiarity: Novel Face, Familiar Face) × 2 (Test Trial: First, Second) repeated measures ANOVA on overall looking times to the primate faces. Participants looked longer overall at the novel than the familiar faces [*F*(1, 23) = 23.75, *p* < 0.001, η^2^ = 0.24], however this main effect of familiarity was qualified by a three-way interaction between species, familiarity, and trial [*F*(1, 23) = 10.66, *p* = 0.003, η^2^ = 0.07], Because participants’ looking at the novel and familiar human and monkey faces differed by test trial, we then conducted four paired samples *t*-tests comparing looking at the novel and familiar human and monkey faces within each test trial. Participants looked significantly longer at the novel compared to the familiar human faces in the first but not the second test trial (First trial: *t*(23) = 5.70, *p* < 0.001 *m*_novel_ = 2648 ms, *sd* = 525; *m*_familiar_ = 1511 ms, *sd* = 501, 95, % *CI*_diff_ = [724, 1550], *d* = 1.16); Second trial: *t*(23) = 1.30, *p* = 0.21, *m*_novel_ = 2165 ms, *sd* = 625; *m*_familiar_ = 1960 ms, *sd* = 435, 95, % *CI*_diff_ = [122, 533], *d* = 0.26)), while participants looked significantly longer at the novel compared to the familiar monkey faces, in the second but not the first test trial (First trial: *t*(23) = 1.47, *p* = 0.15, *m*_novel_ = 2173 ms, *sd* = 501; *m*_familiar_ = 1899 ms, *sd* = 467, 95, % *CI*_diff_ = [111, 660], *d* = 0.30); Second trial: *t*(23) = 3.10, *p* = 0.005, *m*_novel_ = 2409 ms, *sd* = 541; *m*_familiar_ = 1840 ms, *sd* = 432, 95, % *CI*_diff_ = [190, 950], *d* = 0.63)). All tests are corrected for multiple comparisons by adjusting the criterion for statistical significance to an alpha level of 0.01.

#### Time Course of Looking at Human and Monkey Faces

To examine the temporal dynamics of participants’ looking at the novel human and monkey faces during the test trial we used a generalized linear model (GLM) with fixed effects of time, species, and the interaction between time and species. We first divided the data into standard bins of 250 ms, which capture adults’ minimum latency to initiate a saccade (200–250 ms: Yang et al., 2002). We then calculated the proportion looking time at the novel species’ face within each 250 ms bin (0–250 ms, 250–500 ms, 500–750 ms, and 750–1000 ms) and averaged proportions in like bins across trials. We constrained the GLM model to the 1000 ms immediately after presentation of the faces during the first of the two test trials (see Appendix A for complete time course of both test trials). We used effect coding to estimate main effects of time and of the categorical species variable (Human = 1; Monkey = -1) and dummy coding to estimate interactions between species and time (Human = 0; Monkey = 1) with the humans as the reference group. We centered the continuous time variable at bin 1 such that the intercept would represent average looking to the novel faces in bin 1.

**FIGURE 3:**
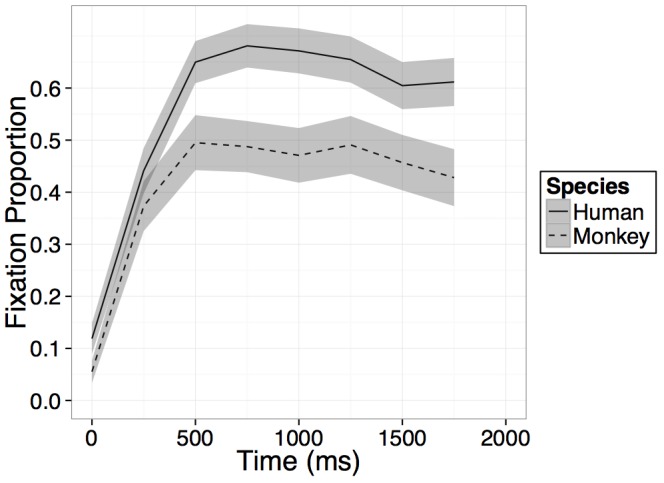
Results. Time course of fixation proportion ± SEM (ribbon) to the Novel face for humans (solid lines) and rhesus monkeys (dashed lines) in the first 2000 ms of the first test trial. GLM is constrained to the first 1000 ms in this window.

Participants increased their looking at the novel face regardless of species on average within the 1000 ms after presentation of the primate faces as evidenced by a main effect of time [*B* = 0.15, *t*(23) = 11.11, *p* < 0.001], however, this main effect of time was qualified by an interaction between time and species [*B* = -0.07, *t*(23) = -2.52, *p* = 0.02], such that on average across the four bins in the model, participants’ rate of looking at the novel human face increased significantly more than their rate of looking at the novel monkey face (see Figure 3). No main effect of species was found and while on average across all bins in the model, participants’ rate of looking at the novel human face increased more than their rate of looking at the novel monkey face, after an initial increase in looking within the first 500–750 ms, looking at both primate faces leveled off or in some cases, began to decrease (see Figure 3).

### Discussion

We re-examined the classic findings of perceptual narrowing for other species’ faces of Pascalis et al. (2002) by testing human adults’ discrimination of frequently encountered human faces and infrequently encountered rhesus monkey faces, using both measures of overall looking as well as the time course of fixations. We also adjusted sample size relative to the original study by using an *a priori* power analyses to determine that *N* = 25 was the appropriate sample size to detect discrimination effects.

Using the same metric which famously showed that adults discriminate between human but not monkey faces (longer looking at the novel face as in Pascalis et al., 2002), we found that adults were able to discriminate both frequent human and infrequent monkey faces. Using a dynamic time course approach testing the time course of adults’ looking at the novel human and monkey faces within the first 1000 ms after initial exposure, we found that adults again discriminated between both human and monkey faces. However, adults’ proportion looking increased more for novel human faces than for novel monkey faces, suggesting a processing advantage for conspecifics.

These two metrics converged to suggest that unimodal perceptual narrowing for faces may not be an obligatory developmental process. However, some issues remain. In Experiment 1, our goal was to replicate the experimental conditions of Pascalis et al. (2002) to make the results directly comparable. This strict replication resulted in an unequal number of human (4) and monkey trials (3). In Experiment 2, we sought to address this potential weakness and also to make the results more generalizable to other primate faces. Thus we conducted a follow-up experiment using faces of a different primate species, Barbary monkeys, with equal numbers of human and monkey faces presented to each participant.

## Experiment 2: Barbary Monkeys

### Materials and Methods

#### Participants

Participants were 22 undergraduate students (*M* age = 20.4 years, *SD* = 3.4; 16 females) at New York University or young adults living in the greater New York City area. All participants gave informed consent and received academic credit or $10 for their participation. All participants had normal or corrected-to-normal vision (20/20). We excluded three additional participants for failure to meet eye-tracking inclusion criteria (See Eye-Tracking Data Reduction and Inclusion Criteria for Experiment 1).

#### Procedure

The procedure of Experiment 2 was identical to that of Experiment 1 with the exception that participants viewed Barbary monkeys instead of rhesus monkeys, and different human faces from a standardized stimulus set. Additionally, participants saw an equal number of human and monkey trial sets (four of each). As in Experiment 1, participant viewed all sets of monkey face trials prior to all sets of human face trials following the procedure of Pascalis et al. (2002). The data are openly available at https://figshare.com/s/f842956676996db1fc2d.

#### Stimuli

Stimuli were 48 color images of human and Barbary monkey (*Macaca sylvanus*) faces. Human and rhesus monkey faces measured 20 cm/18.9 degrees of visual angle vertically and 16 cm/15.2 degrees of visual angle horizontally at a viewing distance of 60sm. Monkey faces were those used in Pascalis et al. (2005) and were obtained courtesy of Dr. Pascalis. Human faces were selected from the NimStim Set of Facial Expressions (Tottenham et al., 2009) and were obtained courtesy of Dr. Tottenham. All images were cropped to a uniform oval, which excluded ears, neck, and hairline using Adobe Photoshop (v. 10.10.1). Images were aligned such that the nose was centered on the common pixel width and the eyes were centered on the common pixel height for all images. Oval shaped AOIs covering each face during experimental trials were 334429 pixels.

#### Apparatus, Eye-Tracking Data Reduction, and Inclusion Criteria

Apparatus, eye-tracking data reduction, and inclusion criteria were identical to those of Experiment 1.

### Results

#### Overall Looking at Human and Monkey Faces

**FIGURE 4:**
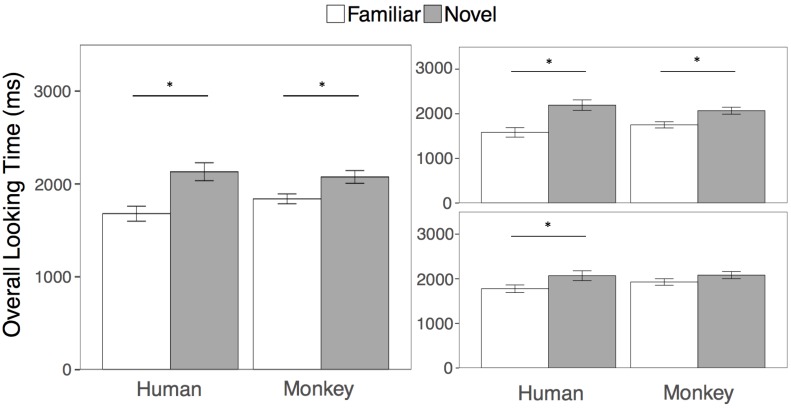
Results. Overall looking times to the Familiar (white bars) and Novel faces (gray bars) in the human and Barbary monkey conditions averaged across both test trials (left), and separately for the first (top right) and second (bottom right) test trials. Error bars represent the standard error of the mean. ^∗^*p* < 0.01.

As in Experiment 1, the goal of the overall looking time analysis was to be directly comparable with the results of Pascalis et al. (2002). We again compared looking at the familiar and novel human and monkey faces in two separate paired-samples *t*-tests collapsed across both test trials. Across test trials 1 and 2, adults looked longer at the novel compared to the familiar face for both humans (*t*(21) = 4.04, *p* = 0.001, *m*_novel_ = 2131 ms, *sd* = 454; *m*_familiar_ = 1680 ms, *sd* = 376, 95, % *CI*_diff_ = [219, 684], *d* = 0.86) and for Barbary monkeys (*t*(21) = 2.94, *p* = 0.008, *m*_novel_ = 2075 ms, *sd* = 322; *m*_familiar_ = 1839 ms, *sd* = 250, 95, % *CI*_diff_ = [69, 403], *d* = 0.62). See Figure 4 for overall looking times to the novel and familiar faces.

We again examined effects of species and trial by conducting a 2 (Species: Human, Monkey) × 2 (Familiarity: Novel Face, Familiar Face) × 2 (Test Trial: First, Second) repeated measures ANOVA on overall looking times to the primate faces. Participants looked longer overall at the novel than the familiar faces [*F*(1, 21) = 20.50, *p* < 0.001, η^2^ = 0.20], with no effect of species or interaction between species and familiarity (all *p*’s > 0.05). However, participants looked longer overall in the first than in the second test trial as evidenced by a main effect of test trial [*F*(1, 21) = 6.16, *p* = 0.02, η^2^ = 0.01]. As in Experiment 1, we conducted four paired samples *t*-tests comparing looking at the novel and familiar human and monkey faces within each test trial. Participants looked significantly longer at the novel compared to the familiar human face in both the first (*t*(21) = 3.18, *p* = 0.004, *m*_novel_ = 2194 ms, *sd* = 549; *m*_familiar_ = 1584 ms, *sd* = 500, 95, % *CI*_diff_ = [212, 1009], *d* = 0.68) and second test trials (*t*(21) = 2.80, *p* = 0.01, *m*_novel_ = 2068 ms, *sd* = 4517; *m*_familiar_ = 1776 ms, *sd* = 401, 95, % *CI*_diff_ = [76, 509], *d* = 0.60). However, participants looked longer at the novel compared to the familiar monkey face in the first (*t*(21) = 2.97, *p* = 0.007, *m*_novel_ = 2070 ms, *sd* = 369; *m*_familiar_ = 1752 ms, *sd* = 325, 95, % *CI*_diff_ = [96, 540], *d* = 0.63) but not the second test trial (*t*(21) = 1.23, *p* = 0.23, *m*_novel_ = 2081 ms, *sd* = 377; *m*_familiar_ = 1927 ms, *sd* = 342, 95, % *CI*_diff_ = [105, 413], *d* = 0.26). We corrected for multiple comparisons by adjusting the criterion for statistical significance to an alpha level of 0.01.

#### Time Course of Looking at Human and Monkey Faces

To examine the temporal dynamics of participants’ looking at the novel human and Barbary monkey faces, we used the same GLM with fixed effects of time, species, and the interaction between time and species as in Experiment 1 (For model details see Experiment 1; for complete time course of both test trials see Appendix B).

**FIGURE 5:**
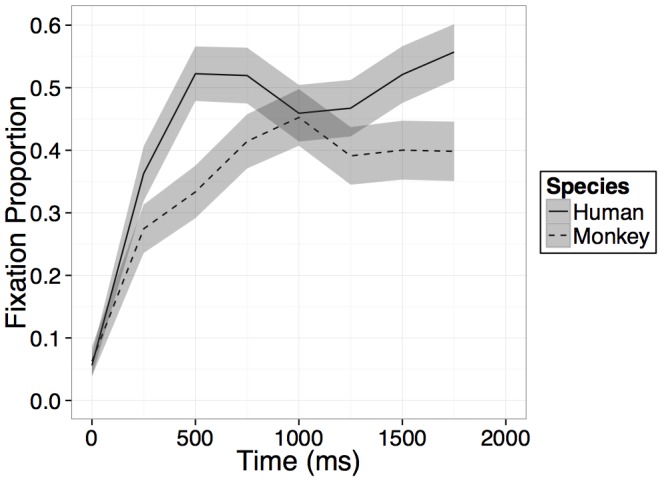
Results. Time course of fixation proportion ± SEM (ribbon) to the Novel face for humans (solid lines) and Barbary monkeys (dashed lines) in the first 2000 ms of the first test trial. GLM is constrained to the first 1000 ms in this window.

Participants increased their looking at the novel face regardless of species on average within the 1000 ms after presentation of the primate faces as evidenced by a main effect of time [*B* = 0.15, *t*(21) = 10.49, *p* < 0.001], and participants looked overall more at the human than the monkey face regardless of time as evidenced by a main effect of species [*B* = -0.04, *t*(21) = -3.22, *p* = 0.004]. However, crucially these effects were again qualified by an interaction between time bin and species [*B* = -0.06, *t*(21) = -2.95, *p* = 0.008], such that, as in Experiment 1, on average across the four bins in the model, participants’ rate of looking at the novel human face increased significantly more than their rate of looking at the novel monkey face (see Figure 5). While on average across all bins in the model, participants’ rate of looking at the novel human face increased more than their rate of looking at the novel monkey face, after an initial increase in looking within the first 500–750 ms, looking at both primate faces leveled off or in some cases, began to decrease (see Figure 5).

### Discussion

In Experiment 2, we sought to further examine visual perceptual narrowing by testing human adults’ discrimination of frequently encountered human faces and infrequently encountered Barbary monkey faces, using both measures of overall looking as well as the time course of fixations. As in Experiment 1, we used an *a priori* power analysis to determine that *N* = 25 was the appropriate sample size to detect discrimination effects. However, unlike in Experiment 1, the goal of which was to replicate the experimental conditions of Pascalis et al. (2002) and therefore make the results directly comparable, we sought to rectify what we thought may be a weakness in the initial design [i.e., unequal numbers of human (4) and monkey (3) trials presented to each participant]. We also sought to test the generalizability of the findings by assessing discrimination of primate faces of other species (Barbary monkeys) and by removing identifying information like ears and hair.

Using the same metric which famously showed that adults discriminate between human but not monkey faces (longer looking at the novel face as in Pascalis et al., 2002), we found that again adults were able to discriminate both frequent human and infrequent monkey faces. Using a dynamic time course approach testing the time course of adults’ looking at the novel human and monkey faces within the first 1000 ms after initial exposure, we found that adults again discriminated between both human and Barbary monkey faces. However, adults’ proportion looking increased more for novel human faces than for novel monkey faces, again suggesting a processing advantage for conspecifics.

As in Experiment 1, these two metrics converged to suggest that unimodal perceptual narrowing for faces may not be an obligatory developmental process. Adults flexibly discriminated between both frequently encountered human and infrequently encountered monkey faces while simultaneously showing a processing advantage for conspecifics.

## General Discussion

Recent findings in unimodal visual perception and multimodal perception have suggested that perceptual narrowing, or diminished sensitivity for infrequently encountered stimuli, might not be an obligatory developmental process (Pascalis et al., 2005; Fair et al., 2012; Uttley et al., 2013; Kubicek et al., 2014; Chien et al., 2016). We tested unimodal visual perception for faces by re-examining the classic findings of perceptual narrowing for faces of Pascalis et al. (2002). We tested human adults’ discrimination of frequently encountered human and infrequently encountered monkey faces. We used both measures of overall looking as well as the time course of fixations to assess discrimination of primate faces. In two experiments, we found evidence that human adults discriminate human and monkey faces using two different measures: longer looking at the novel face using an overall looking approach (as in Pascalis et al., 2002) and increase in looking at the novel face in the first 1000 ms of test trials using a dynamic time course approach. Overall patterns of looking as well as the time course of fixations converged to suggest that unimodal perceptual narrowing for faces may not be an obligatory developmental process. At the same time, humans more readily discriminated human compared to monkey faces in both experiments suggesting an advantage for frequently encountered faces.

Notably, overall looking time patterns in the current study showing successful discrimination of human and monkeys faces are not consistent with previous work showing that adults discriminate between different human but not different monkey faces (Pascalis and Bachevalier, 1998; Pascalis et al., 2002; Dufour and Petit, 2010). These inconsistencies may be attributed to differences in statistical power. *Post hoc* power analyses showed that with a total sample size of *N* = 11 participants, Pascalis et al. (2002) was at 15% observed power to detect a small effect (0.2), 46% for a medium effect (0.5), and 79% for a large effect (0.8; G^∗^Power v 3.1; Faul et al., 2007). Sample sizes from comparable studies were similarly small, ranging from *N* = 9 to *N* = 12 participants (Pascalis and Bachevalier, 1998; Dufour and Petit, 2010). Prior findings in adults may have been statistically underpowered to detect effects of discrimination of monkey faces, which are attenuated compared to discrimination of human faces (see Cohen’s *d* for Experiments 1 and 2). Moreover, previous work also used unequal numbers of human (4) and monkey (3) trials, which makes direct comparison of the discrimination effects across species problematic (Pascalis et al., 2002). In the present studies, we aimed to rectify both of these issues by obtaining a sample large enough to detect an effect of medium size at 80% power (*N* = 25) and by using equal numbers of human and monkey trials in Experiment 2. Given that adults look statistically longer at the novel compared to the familiar monkey face across two species of monkeys (rhesus and Barbary) and in varying ecological conditions (ears, neckline, and hair exposed or removed; white background or gray background; varying size), we suggest that human adults can indeed discriminate between different monkey faces, though this effect is attenuated compared to discrimination for human faces, and that previous findings taken in support of visual perceptual narrowing for primate faces may have been an artifact of the small sample size and coarse-grained looking time task.

Whereas overall looking time analyses suggested that adults discriminate both human and monkey faces, dynamic processing of the two species differed: adults’ proportion looking increased more for the novel human face than the novel monkey face, both for rhesus monkey faces in Experiment 1 and Barbary monkey faces in Experiment 2, suggesting a processing advantage for conspecifics (same-species) compared to other non-human primates. These results parallel findings in multimodal perception in which 12 months old infants are faster to match speech to human faces than they are to match monkey vocalizations to monkey faces, though they match both types of vocalizations to the corresponding species’ face (unpublished data from our laboratory) and in unimodal auditory perception in which adults can correctly identify the valence of both human and some animal vocalizations but are more reliable for humans (Scheumann et al., 2014).

Moreover, a processing advantage for signals produced by conspecifics, while preserving the ability to process signals of related species has also been observed in other animals, for example zebra finches (*Taeniopygia guttata*; Araki et al., 2016), killer whales (*Orcinus orca;* Musser et al., 2014), and sheep (*Ovis aries*; Kendrick et al., 2001; Peirce et al., 2001). In finches for example, some aspects of vocal learning appear innate (e.g., temporal gap coding), but other aspects appear experience-dependent (e.g., syllable morphology) and allow zebra finches to process signals of closely related species (Araki et al., 2016). Similarly, while sheep are able to recognize human faces at similar, though slightly diminished rates compared to conspecific faces, they use distinct processing strategies (Kendrick et al., 2001). Thus some organisms appear to maintain the ability to process signals of related species, though they enjoy a processing advantage for conspecifics. While it is unclear whether a mutual, shared mechanism underlies these processes across species, we speculate that distinct neural mechanisms underlie processing for conspecifics compared with close animal relatives, and that this flexibility may only be evident in species with complex social structures and/or advanced communication systems.

The flexibility that humans demonstrate in unimodal visual and multimodal perception by maintaining sensitivity to distinctions and correspondences between infrequently encountered stimuli, may not extend to speech. Narrowing for speech perception and production has been observed for consonants (Werker and Tees, 1984; Kuhl et al., 1992) and vowels (Polka and Werker, 1994), in infants, children, and adults (Werker and Tees, 1984; Sundara et al., 2006), in monolinguals and bilinguals (Burns et al., 2007), and across many language contrasts (for review see Curtin and Werker, 2007). Moreover, even with explicit training, adult speakers’ perceptual and productive language abilities are resistant to non-native contrasts (Strange and Dittman, 1984; Logan et al., 1991; Pisoni and Lively, 1995). Perceptual narrowing for speech seems more robust to perturbations across stimuli, experimental techniques, or analytic approaches whereas narrowing for unimodal visual and multimodal perception appears not to be an obligatory developmental process. Perceptual narrowing may be specific to the domain of speech and may be one of the ways in which language acquisition in humans is distinct from other types of learning.

Contrary the classic finding (Pascalis et al., 2002), we find that human adults maintain the ability to discriminate between both human and monkey faces, and suggest that a loss of perceptual sensitivity to infrequently encountered images may not be an obligatory developmental process in vision. While adults maintain the ability to discriminate between both frequently and infrequently encountered faces, they show a processing advantage for conspecifics. This processing advantage for conspecifics may be consistent with a perceptual learning account (i.e., that perception for frequently encountered stimuli is enhanced in development, as opposed to perception for infrequent stimuli experiencing a loss or decline). However, data from younger participants would be required to examine perceptual learning directly. Regardless, the present findings support the suggestion that perceptual narrowing is more flexible than has previously been conceptualized, and should be characterized as a refinement or learning and not a loss of ability (Fair et al., 2012; Maurer and Werker, 2013; Flom, 2014; Chien et al., 2016). Perceptual narrowing thus may not be domain-general as has been suggested (Scott et al., 2007). Future research should continue to explore the bounds of perceptual narrowing using a diverse set of experimental and analytic techniques.

Perceptual narrowing has been proposed to be a ubiquitous and obligatory feature of perceptual development. We present data that challenge this account of perceptual development. We find that human adults were able to discriminate between infrequently encountered monkey faces, as well as frequently encountered human faces, suggesting that adults maintain their ability to discriminate between some infrequently encountered visual stimuli. At the same time, adults more readily discriminate between human compared to monkey faces, paralleling conspecific or same-species processing advantages seen in the broader animal literature. Perceptual narrowing should be considered a refinement of abilities and not a complete loss. That adults can discriminate between both frequently and infrequently encountered faces suggests that perceptual narrowing cannot be a domain general, obligatory developmental process.

## Ethics Statement

This study was carried out in accordance with the recommendations of the Guidelines for Research with Human Subjects, the New York University Committee on Activities Involving Human Subjects with written informed consent from all subjects. All subjects gave written informed consent in accordance with the Declaration of Helsinki. The protocol was approved by the New York University Committee on Activities Involving Human Subjects.

## Author Contributions

AS designed experimental protocols, collected data, conducted analyses, wrote and edited manuscript, and designed and edited figures and tables. AV assisted with design of experimental protocols, and writing and editing of manuscript.

## Conflict of Interest Statement

The authors declare that the research was conducted in the absence of any commercial or financial relationships that could be construed as a potential conflict of interest.

## Figures and Tables

**FIGURE A1:**
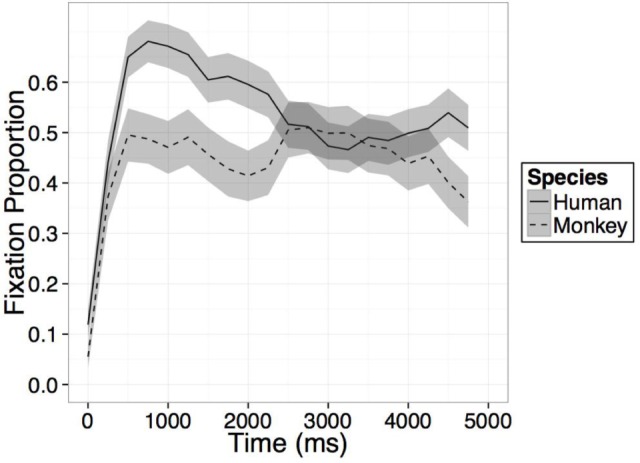
Time course of fixation proportion ± SEM (ribbon) to the Novel face for humans (solid lines) and rhesus monkeys (dashed lines) in the first test trial. GLM in Experiment 1 is constrained to the first 1000 ms in this window.

**FIGURE A2:**
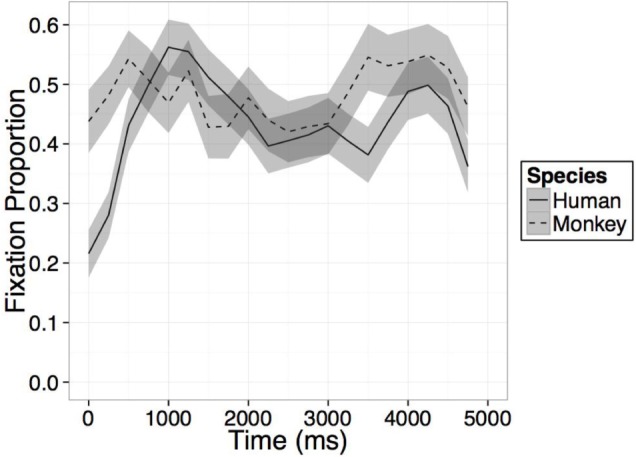
Time course of fixation proportion ± SEM (ribbon) to the Novel face for humans (solid lines) and rhesus monkeys (dashed lines) in the second test trial.

**FIGURE B1:**
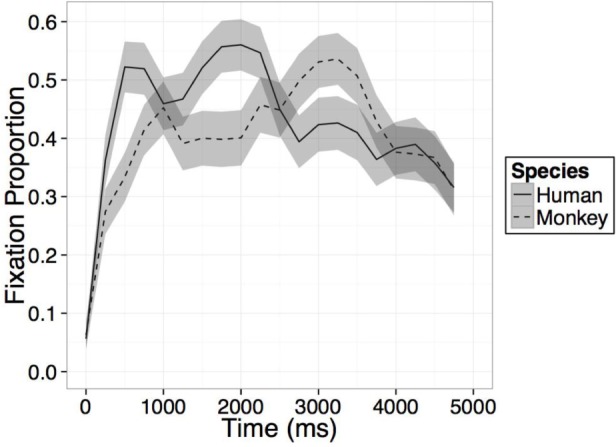
Time course of fixation proportion ± SEM (ribbon) to the Novel face for humans (solid lines) and Barbary monkeys (dashed lines) in the first test trial. GLM in Experiment 2 is constrained to the first 1000 ms in this window.

**FIGURE B2:**
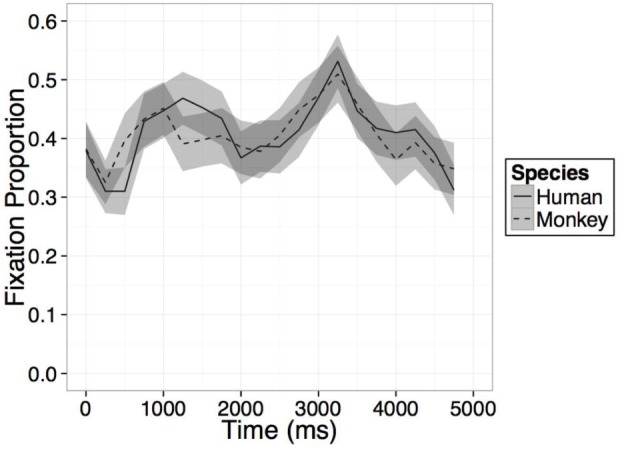
Time course of fixation proportion ± SEM (ribbon) to the Novel face for humans (solid lines) and Barbary monkeys (dashed lines) in the second test trial.

## Footnotes

1 www.smivision.com/
